# The Constituents of Roots and Stems of *Illigera luzonensis* and Their Anti-Platelet Aggregation Effects

**DOI:** 10.3390/ijms150813424

**Published:** 2014-07-31

**Authors:** Chieh-Hung Huang, Yu-Yi Chan, Ping-Chung Kuo, Yu-Fon Chen, Ren-Jie Chang, Ih-Sheng Chen, Shwu-Jen Wu, Tian-Shung Wu

**Affiliations:** 1 Department of Chemistry, National Cheng Kung University, Tainan 70101, Taiwan; E-Mails: hjh2000.tw@gmail.com (C.-H.H.); l3894116@mail.ncku.edu.tw (R.-J.C.); 2 Department of Biotechnology, Southern Taiwan University of Technology, Tainan 71005, Taiwan; E-Mail: yuyichan@mail.stust.edu.tw; 3 Department of Biotechnology, National Formosa University, Yunlin 63201, Taiwan; E-Mail: pcckuoo@sunws.nfu.edu.tw; 4 Department of Life Sciences, National Cheng Kung University, Tainan 70101, Taiwan; E-Mail: yufons@gmail.com; 5 Department of Pharmacy, Kaohsiung Medical University, Kaohsiung 80708, Taiwan; E-Mail: m635013@kmu.edu.tw; 6 Department of Medical Laboratory Science and Biotechnology, Chung Hwa University of Medical Technology, Tainan 71703, Taiwan; 7 Department of Pharmacy, National Cheng Kung University, Tainan 70101, Taiwan; 8 Department of Pharmacy and Graduate Institute of Pharmaceutical Technology, Tajen University, Pingtung 90741, Taiwan

**Keywords:** *Illigera luzonensis*, aporphine, alkaloid, benzenoid, anti-platelet aggregation effect

## Abstract

Phytochemical investigation of the roots and stems of *Illigera luzonensis* afforded two new aporphine alkaloids (**1**) and (**2**), one new bisdehydroaporphine alkaloid (**3**), and one new benzenoid (**4**), along with 28 known structures. The structures of new compounds were elucidated by spectral and MS analysis. Among the isolated compounds, (**1**) and (**4**–**13**) were subjected into the examination for their inhibitory effects on the aggregation of washed rabbit platelets.

## 1. Introduction

*Illigera luzonensis* Merr. (Hernandiaceae) is a scandent shrub distributed in the Luzon (Philippines), Ryukyus, Pulau Palawan, and South Taiwan [1]. The Hernandiaceae family was reported to contain aporphines, noraporphines, oxoaporphines, lignans, benzylisoquinolines, and their derivatives [2,3,4,5]. Many of these isolated compounds displayed some biological activities, including anti-platelet aggregation, anti-plasmodial, vasorelaxing, cytotoxic, and antioxidant effects [4,5]. According to the previous literature, aporphines and oxoaporphines were isolated from the roots and stems of *I. luzonensis* and showed cytotoxic activities *in vitro* [6,7,8,9]. Due to the notorious anti-platelet aggregation bioactivity of the titled species, in the present study we aimed to investigate the roots and stems of *I. luzonensis.* Four new compounds (**1**–**4**) were characterized. In addition, the isolated compounds were studied for their inhibitory effects on the aggregation of washed rabbit platelets.

## 2. Results and Discussion

### 2.1. Purification and Characterization

The 85% aqueous MeOH extract of the roots and stems of *I. luzonensis* was suspended in H_2_O and partitioned with CHCl_3_ to afford CHCl_3_ and H_2_O soluble layers, respectively. Each layer was subjected into purification by a combination of conventional chromatographic techniques to result in four new compounds (**1**–**4**). In addition, 27 known compounds were identified to be methyl ferulate (**5**) [10], (6-methoxy-9*H*-β-carbolin-1-yl)-(4-methoxy-phenyl)-methanone (**6**) [11], cathafiline (**7**) [12], methyl *p*-hydroxycinnamate (**8**) [13], 2-(4'-hydroxyphenyl)-ethyl tricosanoate (**9**) [14], machigline (**10**) [15], launobine (**11**) [7], actinodaphnine (**12**) [16], ferulic acid (**13**) [17], (+)-*N*-methoxycarbonyl-lindcarpine [18], caaverine [19], noroliveroline [20], pallidine [21], thalifoline [22], nicotinic acid [22], *p*-hydroxybenzaldehyde [23], *p*-hydroxybenzoic acid [24], methylparaben [25], vanillin [26], vanillic acid [27], methyl vanillate [22], methyl caffeate [28], methyl syringate [29], *p*-hydroxybenzyl methyl ether [30], squalene [31], β-sitosterol [32], and allantoin [33] by comparison of their physical and spectral data with those reported in the literature.

### 2.2. Structural Elucidation of Compounds **1**–**4**

**Figure 1:**
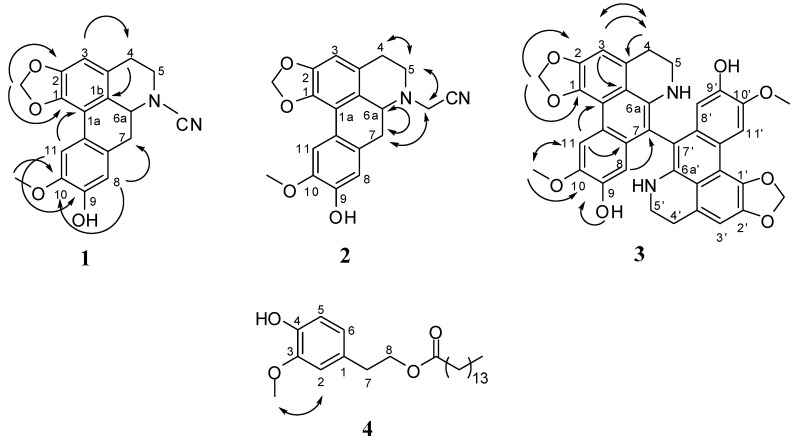
Selected HMBC (→) and NOESY (↔) spectrum for compounds **1**–**4**.

Compound **1** was obtained as optically active syrup. The HREIMS of **1** showed a molecular ion peak at *m*/*z* 336.1113 corresponding to the molecular formula C_19_H_16_N_2_O_4_ and was also corroborated by ^13^C NMR spectrum which displayed 19 carbon signals. The UV spectrum exhibited absorption maxima at 220, 233 (sh), 274 (sh), 282, 308, and 316 (sh) nm was typical of the occurrence for the basic skeleton of aporphine with 1,2,9,10-tetraoxygenation [34]. The IR spectrum of **1** showed a hydroxy absorption at 3352 cm^−1^, a nitrile group at 2214 cm^−1^ which was also proved by ^13^C-NMR (δ 110.2), and two methylenedioxy absorptions at 1055 and 948 cm^−1^, respectively. In the ^1^H NMR spectrum of **1** (Table 1), it displayed the typical aromatic proton singlets at δ 7.63, 6.85, and 6.53 corresponding to a 1,2,9,10-tetrasubstituted aporphine alkaloid which was assigned to the H-11, H-8, and H-3 [34]. There were also two *gem*-coupling doublets at δ 6.11 (1H, *J* = 0.8 Hz) and δ 5.96 (1H, *J* = 0.8 Hz) characteristic for a methylenedioxy group, a D_2_O exchangeable broad singlet at δ 5.71 (1H) for a hydroxy group, and a singlet at δ 3.99 (3H) for a methoxy group. The HMBC experiment (Figure 1) showed long-range correlations from the methoxy group to the carbon signal at C-10; and from the methylenedioxy signals (δ 6.11 and 5.96) to the carbon signals at C-1 and C-2, respectively. In addition, three mutually coupling aliphatic proton signals at δ 4.25 (1H, dd, *J* = 14.0, 4.8 Hz), 3.12 (1H, dd, *J* = 14.0, 4.8 Hz), and 2.92 (1H, t, *J* = 14.0 Hz) were assigned as H-6a, H-7e, and H-7a according to their chemical shifts and coupling constants. The stereochemistry of H-6a was determined as α due to the positive specific rotation of **1** [35]. Extensive interpretation of COSY, NOESY, HMQC and HMBC experimental data of **1** established all the connectivity, including the sites of the attachment of the methoxy, hydroxy, and methylenedioxy groups, to accomplish the full assignment of all ^1^H and ^13^C NMR signals (Table 1). On the basis of the foregoing spectral studies, the structure of **1** was determined as (*S*)-*N*-nitrile-9-hydroxy-1,2-methylenedioxy-10-dimethoxy-5,6,6a,7-tetrahydro-4*H*-dibenzo[de,g]quinoline and trivially named as illigeluzine A.

Compound **2** was afforded as optically active syrup. The HREIMS of **2** showed a molecular ion peak at *m*/*z* 350.1270 corresponding to the molecular formula C_20_H_18_N_2_O_4_, which was one CH_2_ unit more than that of **1**. The UV absorption maxima, IR absorption bands, ^1^H and ^13^C NMR spectra of **2** were very similar to those of **1**. The only differences were one more aliphatic methylene group at δ 4.09 (1H, d, *J* = 17.6 Hz) and 3.73 (1H, d, *J* = 17.6 Hz) in **2**. The location of this methylene unit was attached at the nitrogen atom according to the NOESY spectrum interpretation, in which correlations were found between δ 3.12 (H-5) and δ 4.09, and between δ 2.90 (H-7) and δ 4.09, respectively. The stereochemistry of H-6a was also determined as α due to the positive specific rotation of **2** [35]. Conclusively, the chemical structure of **2** was determined as (*S*)-*N*-acetonitrile-9-hydroxy-1,2-methylenedioxy-10-dimethoxy-5,6,6a,7-tetrahydro-4*H*-dibenzo[de, g]quinoline and trivially named as illigeluzine B.

Compound **3** was purified as brown needles, with mp > 280 °C. The FABMS of **3** showed one pseudomolecular ion and one molecular ion peaks at *m*/*z* 617 and 616, which implied the presence of a dimeric aporphine alkaloid. The UV spectrum exhibited absorption maxima at 204, 268, 332, and 393 nm was typical of the occurrence for the basic skeleton of dehydro-aporphine alkaloid with 1,2,9,10-tetraoxygenation [34]. The IR spectrum of **3** showed a hydroxy and amino absorption band at 3382 cm^−1^, and two methylenedioxy absorption band at 1056, and 952 cm^−1^. In the ^1^H NMR spectrum of **3**, the characteristic aromatic singlets at δ 8.42, 7.07, and 6.34 corresponding to a 1,2,9,10-tetrasubstituted dehydro-aporphine alkaloid were assigned to be the H-11, -11', -3, -3', -8, and -8'. It also displayed a methylenedioxy group at δ 6.28 (4H, s), a D_2_O exchangeable hydroxy group at δ 9.07 (2H, br s), a D_2_O exchangeable amino group δ 4.54 (2H, s), and a methoxy group at δ 3.84 (6H, s), respectively. The significant spectral characteristics of **3** were the disappearances of H-7 and H-6a. The HMBC experiment (Figure 1) exhibited a ^3^*J*-correlation between H-8, -8' (δ 3.99) and C-7, -7', and it suggested that C-7 and -7' were quaternary carbon atoms. According to the molecular formula and the HMBC spectral analysis, the structure of 3 could be defined as a symmetric dimer of dehydroaporphine connected through C-7 and C-7'. Comprehensive interpretation of all the COSY, NOESY, HMQC and HMBC spectra of **3** established all the connectivity, including the location of the methoxy, hydroxy, and methylenedioxy groups, to accomplish the complete assignment of all ^1^H and ^13^C NMR signals. Therefore, the chemical structure of **3** was concluded as bisdehroactinodaphnine as shown in Figure 1.

**Table 1:** ^1^H and ^13^C NMR data of compounds **1** and **2** ^a^.

Position	1		2
	d_H_ (J, Hz)	*d*_C_	*d*_H_ (*J*, Hz)
1		142.2	
1a		116.7	
1b		125.2	
2		147.4	
3	6.53, s	107.0	6.52, s
3a		123.2	
4	3.10, m	28.1	2.91, m
	2.74, dt (16.2, 3.8)		2.68, br d (15.6)
5	3.43, td (12.6, 3.8)	47.2	3.12, m
	3.67, m		3.00, dd (11.2, 5.2)
6a	4.25, dd (14.0, 4.8)	54.9	3.62, br d (13.8)
7	2.9, t (14.0)	33.7	
	3.12, dd (14.0, 4.8)		2.90, br d (13.8)
7a		127.0	2.59, t (13.8)
8	6.85, s	114.5	6.82, s
9		145.5	
10		145.7	
11	7.63, s	110.0	7.63, s
11a		122.5	
OH-9 ^b^	5.71, br s		5.71, br s
OCH_3_-10	3.99, s	56.2	4.04, s
–OCH_2_O–	5.96, d (0.8)	100.9	5.94, d (1.8)
	6.11, d (0.8)		6.09, d (1.8)
–CH_2_CN			4.09, d (17.6)
			3.73, d (17.6)
CN		110.2	

^a^ ^1^H NMR data were measured at 400 MHz for **1**, and 200 MHz for **2**. ^13^C NMR data were measured at 100 MHz for **1**. The assignments are based on ^1^H–^1^H COSY, NOESY, HMQC and HMBC spectra; ^b^ D_2_O exchangeable.

Compound **4** was isolated as white powder. The ESIMS of **4** displayed a molecular ion peak at *m*/*z* 392. The UV spectrum exhibited absorption maxima at 228 and 281 nm was typical of the occurrence for the basic skeleton of benzenoid [36]. The IR spectrum showed hydroxy and ester groups at 3457 and 1737 cm^−1^. The ^1^H NMR characteristics including δ 6.80 (1H, d, *J* = 1.8 Hz), 6.78 (1H, d, *J* = 8.2 Hz), and 6.68 (1H, dd, *J* = 8.2, 1.8 Hz) indicated the presence of 1,2,4-trisubstituted aromatic ring system. It also showed a D_2_O exchangeable hydroxy signal at δ 5.91 (1H, br s) and a methoxy signal at δ 3.86 (3H, s) which displayed NOESY correlation with H-2. In addition, two mutually coupling aliphatic methylene groups at δ 4.23 (2H, t, *J* = 7.2 Hz) and 2.83 (2H, t, *J* = 7.2 Hz), one methylene connected with carbonyl group at δ 2.28 (2H, t, *J* = 7.6 Hz), one terminal methyl group at δ 0.88 (3H, t, *J* = 6.8 Hz), and one set of long-chain alkyl methylene groups at δ 1.26 (24H, br s) constructed the phenylethyl alkanoate basic structure. Detailed analysis of the COSY and NOESY spectral data of **3** furnished the full assignment of all ^1^H-NMR signals. Consequently, the structure of **4** was determined as 4-hydroxy-3-methoxyphenethyl pentadecanoate and it was named trivially as illigeraol A.

### 2.3. Anti-Platelet Aggregation Evaluation Bioassay

Platelets play a pivotal role in development of cardiovascular disease [37]. Arterial thrombosis is the acute complication that develops on the chronic lesions of atherosclerosis and reasons heart attack and stroke. These chronic inflammatory processes are the central pathophysiological mechanism largely driven by lipid accumulation, and provide the substrate for occlusive thrombus formation. Most current models of thrombus development propose a key role for collagen (and possibly vessel wall-derived thrombin) in initiating platelet activation in primary adherent platelets, whereas subsequent propagation of thrombin (platelet aggregation) is primarily driven by agonists released or generated from the platelet surface, including ADP, TXA2 (Thromboxane A2) and thrombin [38].

Platelets circulate in the blood of mammals and are involved in hemostasis, leading to the formation of blood clots. Too many platelets form blood clots that may obstruct blood vessels and induce strokes, myocardial infarctions, and pulmonary embolisms. Sometimes this situation also results in the blockage of blood vessels to other parts of the body, including the extremities of the arms or legs [39]. The traditional medicinal use of *Illigera luzonensis* is to promote the blood circulation necessary for removing blood stasis. Therefore, the purified compounds were examined for their anti-platelet aggregation bioactivity. However, due to the limited quantity of the purified compounds, only inhibitory effects on the aggregation of washed rabbit platelets were investigated. The anti-platelet aggregation effects are summarized in Table 2 and Table 3. Among the tested compounds, **1**, **5**, **6**, **7**, **8**, and **11**–**13** displayed significant inhibitory effects on the aggregation of washed rabbit platelets stimulated by arachidonic acid (AA). Compounds **4**, **9**, and **10** did not inhibit the rabbit platelet aggregation significantly, and therefore the data was not included in Table 2. Compounds **3**, **5**, and **8** were found to be the most effective compounds among the tested, with IC_50_ values in the range of 0.5 and 0.2 μg/mL.

Generally the significant inhibitory effects on the aggregation of washed rabbit platelets were related to the aporphine alkaloids; however, in the present study the most potent compounds **5** and **8** were benzenoids. However, the aporphine alkaloids **1**, **7**, and **10**–**12** still exhibited moderate antiplatelet aggregation bioactivity. On the other hand, the activities of these purified compounds against thrombin (Thr) and collagen (Col) induced aggregation were not as effective, with the exceptions of **1**, **5**–**8**, **12**, and **13** at 100 μg/mL. Platelet activating factor (PAF) is a potent phospholipid activator and mediator of many leukocyte functions, including platelet aggregation and degranulation, inflammation, and anaphylaxis. In the present examination, **5**, **7**, and **12** displayed significant inhibitory effects on the aggregation of platelets stimulated by PAF. Actinodaphnine (**12**), which was belonged to the aporphine alkaloid, exhibited the most effective inhibition on the aggregation of washed rabbit platelets with IC_50_ value in the range of 50 and 20 μg/mL.

## 3. Experimental Section

### 3.1. General

The UV spectra were obtained with Hitachi UV-3210 spectrophotometer. The IR spectra were measured with a Shimadzu FTIR Prestige-21 spectrometer (Shimadzu, Kyoto, Japan). Optical rotations were recorded with a Jasco DIP-370 digital polarimeter (Jasco, Tokyo, Japan) in a 0.5 dm cell. The ESIMS and HRESIMS were taken on a Bruker Daltonics APEX II 30e spectrometer (Bruker, Billerica, MA, USA). The FABMS and HRFABMS were taken on a Jeol JMS-700 spectrometer (Jeol, Peabody, MA, USA). The ^1^H and ^13^C NMR spectrums were measured by Bruker Avance 200 and 400 NMR spectrometers (Bruker) with TMS as the internal reference, and chemical shifts are expressed in δ (ppm). Sephadex LH-20, silica gel (70–230 and 230–400 mesh; Merck, Darmstadt, Germany) and reversed-phase silica gel (RP-18; particle size 20–40 μm; Silicycle, Quebec, QC, Canada) were used for column chromatography, and silica gel 60 F_254_ (Merck) and RP-18 F_254S_ (Merck) were used for TLC. HPLC was performed on a Shimadzu LC-10AT_VP_ (Shimadzu) system equipped with a Shimadzu SPD-M20A diode array detector at 250 nm, (Shimadzu) a Purospher STAR RP-8e column (5 µm, 250 × 4.6 mm, Merck Millipore, Billerica, MA, USA) and Cosmosil 5C_18_ ARII (250 × 4.6 mm i.d. Nacalai Tesque Inc., Kyoto, Japan).

### 3.2. Plant Materials

The whole plants of *Illigera luzonensis* Merr. were collected from Pingtung, Taiwan in January 1995. The plant was authenticated by Professor C.S. Kuoh, Department of Life Science, National Cheng Kung University, Taiwan. The voucher specimens (DG-199) have been deposited in the Department of Chemistry, National Cheng Kung University, Tainan, Taiwan.

### 3.3. Extraction and Isolation

The roots and stems of plant materials (11.9 kg) were cut into small pieces and heated at refluxed with 85% aqueous MeOH (7 × 80 L). The resulting MeOH extract (355 g) was partitioned between CHCl_3_ and H_2_O (each 3L) for five times to yield the CHCl_3_ layer (160 g) and H_2_O layer (195 g). The CHCl_3_ layer was subjected to silica gel column chromatography (CC) using a gradient mixture of CHCl_3_–MeOH (25:1, 19:1, 9:1, 7:1, 5:1, 3:1, 1:1) as eluent to give 9 fractions (Fr. 1–9). Fr. 4 was purified by column chromatography over silica gel (*n*-hexane-methanol 19:1) to yield bisdehydroactinodaphnine (24.7 mg), (6-methoxy-9*H*-β-carbolin-1-yl)-(4-methoxy-phenyl)-methanone (17.6 mg), 2-(4'-hydroxyphenyl)-ethyl tricosanoate (10.2 mg), *p*-hydroxy-benzaldehyde (6.3 mg), *p*-hydroxybenzoic acid (3.2 mg), methyl-paraben (4.9 mg), vanillin (40.9 mg), methylvanillate (12.5 mg), methyl *p*-hydroxycinnamate (7.7 mg), methyl ferulate (9.1 mg), *p*-hydroxybenzyl methyl ether (11.4 mg), squalene (13.8 mg), β-sitosterol (1.23 mg), illigeraol A (30.2 mg). Fr. 5 was subjected to chromatography on silica gel (CHCl_3_–MeOH, 9:1) to yield vanillic acid (16.9 mg). Fr. 6 was chromatographed over silica gel (CHCl_3_–MeOH, 9:1) to yield launobine (12.8 mg), caaveine (2.5 mg), noroliveroline (1.6 mg), pallidine (6.7 mg), methyl caffeate (7.5 mg). Fr. 8 was purified by CC over silica gel (CHCl_3_–MeOH, 9:1) to yield actinnodaphnine (2.62 g), machigline (3.35 g), cathafiline (8.4 mg), (+)-*N*-methoxylcarbonyl-nandigerine (1.6 mg), illigeluzine A (10.4 mg), illigeluzine B (0.7 mg), thalfoline (5.4 mg), nicotinic acid (3.7 mg), and methyl syringate (24.5 mg). The H_2_O layer (195 g) was filtered and recrystallized to yield allantoin (3.34 g). All the other residues did not afford any compounds.

#### 3.3.1. Illigeluzine A (**1**)

Yellow syrup; [α]_D_ +31° (c 0.04, MeOH); UV (MeOH) λ_max_ 315.6 (sh), 307.6, 282.4 (sh), 274.4, 233.2 (sh), 220.0 nm; IR (KBr) ν_max_ 3352, 2214, 1600, 1508, 1460, 1278, 1107, 1055, 948, 871 cm^−1^; ^1^H and ^13^C NMR see Table 1; EIMS *m*/*z* (*rel*. *int*.) 336 (M^+^, 99), 282 (32), 281 (100), 111 (23), 97 (38), 95 (26), 83 (38); HREIMS *m*/*z* 336.1113 [M]^+^ (Calcd for C_19_H_16_N_2_O_4_, 336.1110).

#### 3.3.2. Illigeluzine B (**2**)

Yellow syrup; [α]_D_ +68° (c 0.007, MeOH); UV (MeOH) λ_max_ 314.0 (sh), 306.4, 281.2, 272.0 (sh), 234.0, 217.6 nm; IR (KBr) ν_max_ 3480, 2235, 1605, 1523, 1392, 1237, 1100, 1051, 950, 786 cm^−1^; ^1^H NMR see Table 1; EIMS *m*/*z* (*rel*. *int*.) 350 (M^+^, 74), 349 (70), 324 (67), 323 (67), 310 (44), 308 (41), 282 (46), 97 (45), 83 (48), 71 (54), 69 (58), 57 (100), 55 (65); HREIMS *m*/*z* 350.1270 [M]^+^ (Calcd for C_20_H_18_N_2_O_4_, 350.1267).

**Table 2:** Antiplatelet aggregation effects of **1**, **4**, and **5**–**7**.

Aggregation (%)						
Inducer	Control	Conc. (μg/mL)	1	5	6	7
AA (100 μM)	90.2 ± 0.8	100	0.0 ± 0.0 ****	0.0 ± 0.0 ****	0.0 ± 0.0 ****	0.0 ± 0.0 ****
		50	4.7 ± 2.8 ****	–	5.8 ± 5.2 ****	0.0 ± 0.0 ****
		20	48.0 ± 13.7 ***	–	24.9 ± 14.1 ****	16.5 ± 11.1 ****
		10	78.7 ± 4.2 ***	–	68.4 ± 8.6 ***	49.9 ± 14.2 ***
		5	85.6 ± 3.2 *	–	86.9 ± 1.2 ***	83.8 ± 2.0 ***
		2	–	0.0 ± 0.0 ****	–	–
		1	–	17.8 ± 11.4 ****	–	–
		0.5	–	30.6 ± 13.5 ****	–	–
		0.2	–	83.6 ± 2.5 ***	–	–
Thr (0.1 U/mL)	94.5 ± 1.0	100	90.9 ± 2.1 *	89.5 ± 3.2 *	92.4 ± 2.1	80.3 ± 2.6 ***
		50	–	–	–	–
		20	–	–	–	–
Col (10 μM)	85.6 ± 0.8	100	6.8 ± 2.1 ****	0.0 ± 0.0 ****	9.5 ± 1.0 ****	9.5 ± 1.0 ****
		50	–	–	–	–
		20	–	–	–	–
PAF (2 ng/mL)	84.9 ± 0.6	100	57.5 ± 5.1 ****	0.0 ± 0.0 ****	64.3 ± 3.1 ****	0.0 ± 0.0 ****
		50	–	51.5 ± 0.7 ****	–	54.1 ± 2.9 ****
		20	–	71.2 ± 2.1 ****	–	76.4 ± 3.3 **
		10	–	78.1 ± 3.0 **	–	81.5 ± 1.9 *
		5	–	81.6 ± 2.4 *	–	84.3 ± 0.4

Values are means ± SD (*n* = 3–5); *: *p <* 0.05; **: *p <* 0.01; ***: *p <* 0.001; ****: *p <* 0.0001, indicates statistical significance compared to control groups. Abbreviations: AA, arachidonic acid; Thr, thrombin; Col, collagen; PAF, platelet activating factor.

**Table 3:** Antiplatelet aggregation effects of **8**, and **11**-**13**.

Aggregation (%)						
Inducer	Control	Conc. (μg/mL)	8	11	12	13
AA (100 μM)	90.2 ± 0.8	100	0.0 ± 0.0 ****	–	0.0 ± 0.0 ****	0.0 ± 0.0 ****
		50	–	25.7 ± 12.4 ****	0.0 ± 0.0 ****	–
		20	–	43.4 ± 17.2 ***	11.9 ± 10.6 ****	–
		10	–	62.6 ± 15.2 **	54.3 ± 14.4 ***	–
		5	0.0 ± 0.0 ****	70.5 ± 14.1 *	85.1 ± 3.9 *	0.0 ± 0.0 ****
		2	4.8 ± 2.6 ****	88.0 ± 2.4	90.2 ± 0.8	43.0 ± 12.3 ****
		1	30.2 ± 13.5 ****	–	–	77.3 ± 8.6 *
		0.5	43.8 ± 16.4 ***	–	–	–
		0.2	84.1 ± 2.4 ***	–	–	–
Thr (0.1 U/mL)	94.5 ± 1.0	100	86.6 ± 0.8 ****	–	12.5 ± 7.7 ****	87.8 ± 2.1 **
		50	–	90.5 ± 0.8 **	–	–
		20	–	–	–	–
Col (10 μM)	85.6 ± 0.8	100	0.0 ± 0.0 ****	–	7.1 ± 5.8 ****	5.6 ± 1.1 ****
		50	–	71.3 ± 4.6 **	–	–
		20	–	–	–	–
PAF (2 ng/mL)	84.9 ± 0.6	100	56.3 ± 6.4 ***	–	0.0 ± 0.0 ****	71.3 ± 1.1 ****
		50	–	67.0 ± 1.8 ****	0.0 ± 0.0 ****	–
		20	–	–	58.2 ± 2.2 ****	–
		10	–	–	76.7 ± 1.2 ****	–
		5	–	–	82.2 ± 1.9 *	–

Values are means ± SD (*n* = 3–5); *: *p <* 0.05; **: *p <* 0.01; ***: *p <* 0.001; ****: *p <* 0.0001, indicates statistical significance compared to control groups.

#### 3.3.3. Bisdehydroactinodaphine (**3**)

Brown needles; mp > 280 °C; UV (MeOH) λ_max_ 393.2 (3.55), 331.6 (3.85), 268.4 (4.47), 204.0 (4.41) nm; IR (KBr) ν_max_ 3382, 1595, 1461, 1387, 1249, 1146, 1056, 952 cm^−1^; ^1^H-NMR (DMSO-*d*_6_) δ 9.07 (2H, br s, D_2_O exchangeable, –OH), 8.42 (2H, s, H-11, -11'), 7.06 (2H, s, H-3, -3'), 6.34 (2H, s, H-8, -8'), 6.28 (4H, s, (–OCH_2_O–) × 2), 4.54 (2H, br s, D_2_O exchangeable, –NH), 3.84 (6H, s, (–OCH_3_) × 2), 3.22 (4H, br s, H-5, -5'), 3.06 (4H, br s, H-4, -4'); ^13^C-NMR (DMSO-*d*_6_) δ 147.6, 145.0, 144.6, 140.5, 139.2, 129.2, 128.6, 117.4, 117.0, 116.3, 109.9, 108.1, 106.8, 105.1, 100.9, 56.0, 40.7, 30.6; FABMS *m*/*z* (*rel*. *int*.) 617 ([M + 1]^+^, 4), 616 (M^+^, 8), 307 (25), 289 (12), 238 (14), 155 (29), 154 (100), 138 (38), 137 (75), 136 (68), 120 (12), 107 (25).

#### 3.3.4. Illigeraol A (**4**)

White powder; UV (MeOH) λ_max_ 281.2 (3.24), 227.6 (3.56) nm; IR (KBr) ν_max_ 3457, 1737, 1608, 1517, 1467, 1272, 1170, 1026, 810 cm^−1^; ^1^H-NMR (CDCl_3_) δ 6.80 (1H, d, *J* = 1.8 Hz, H-2), 6.78 (1H, d, *J* = 8.2 Hz, H-5), 7.06 (1H, dd, *J* = 8.2, 1.8 Hz,, H-6), 5.91 (1H, br s, D_2_O exchangeable, –OH), 4.23 (2H, t, *J* = 7.2 Hz, H-8), 2.83 (2H, t, *J* = 7.2 Hz, H-7), 2.28 (2H, t, *J* = 7.6 Hz, H-2'), 0.88 (2H, t, *J* = 6.8 Hz, –CH_3_); EIMS *m*/*z* (*rel*. *int*.) 392 (M^+^, 5), 218 (10), 151 (32), 150 (100), 125 (11), 111 (22).

### 3.4. Antiplatelet Aggregatory Bioassay

An assay of the antiplatelet aggregatory activity of the isolated compound was conducted according to the procedures of Teng and coworkers [40,41]. Washed platelets were prepared from blood withdrawn with a siliconized syringe from the marginal vein of New Zealand rabbits. The platelet suspension was obtained from EDTA-anticoagulated platelet-rich plasma according to the washing procedure described previously. The platelet number was determined using a cell counter (Hema-laser 2, Sebia, Molineaux, France) and adjusted to 3.0 × 10^8^ platelets/mL. The platelet pellets were suspended in Tyrode’s solution containing Ca^2+^ (1 mM) and bovine serum albumin (0.35%). All glassware was siliconized. Platelet aggregation was measured using the turbidimetric method [40]. The aggregations were measured with a Lumi-aggregometer (Model 1020, Payton, Stouffville, ON, Canada) connected to two dual-channel recorders.

## 4. Conclusions

In our investigation, the major constituents of the titled plant were aporphine alkaloids and 32 compounds, including 4 new compounds **1**–**4** were characterized from roots and stems of *I. luzonensis.* This is the first report of *N*-nitrile and *N*-acetonitrile aporphine alkaloids from natural sources. In the evaluation of anti-platelet aggregation effects, compounds **5** and **8** were the most effective. These results further indicated that the *Illigera* species are valuable sources for the discovery of natural anti-platelet aggregation lead drugs.
